# Brain, cognitive, and physical disability correlates of decreased quality of life in patients with Huntington’s disease

**DOI:** 10.1007/s11136-022-03220-0

**Published:** 2022-08-17

**Authors:** Estefanía Junca, Mariana Pino, Hernando Santamaría-García, Sandra Baez

**Affiliations:** 1 Universidad de los Andes, Bogotá, Colombia; 2 Universidad Autónoma del Caribe, Barranquilla, Colombia; 3 Pontificia Universidad Javeriana. PhD program of Neuroscience, Bogotá, Colombia; 4 Centro de Memoria y Cognición intellectus, Bogotá, Colombia

**Keywords:** Huntington’s disease, Quality of life, Caregivers, Brain volumes, WHOQOL-BREF

## Abstract

**Purpose:**

Following a case–control design, as a primary objective, this study aimed to explore the relationship between quality of life (QoL) scores and gray matter (GM) volumes in patients with Huntington’s disease (HD). As a secondary objective, we assessed the relationship between QoL scores and other important behavioral, clinical and demographical variables in patients with HD and HD patients’ caregivers.

**Methods:**

We recruited 75 participants (25 HD patients, 25 caregivers, and 25 controls) and assessed their QoL using the World Health Organization Quality of Life scale-Brief Version (WHOQOL-BREF). Participants were also assessed with general cognitive functioning tests and clinical scales. In addition, we acquired MRI scans from all participants.

**Results:**

Our results showed that patients exhibited significantly lower scores in all four QoL domains (physical health, psychological wellbeing, social relationships, and relationship with the environment) compared to caregivers and controls. Caregivers showed lower scores than controls in the physical health and the environmental domains. In HD patients, lower scores in QoL domains were associated with lower GM volumes, mainly in the precuneus and the cerebellum. Moreover, in HD patients, physical disability and GM volume reduction were significant predictors of QoL decrease in all domains. For caregivers, years of formal education was the most important predictor of QoL.

**Conclusions:**

HD patients exhibit greater GM volume loss as well as lower QoL scores compared to caregivers and controls. However, caregivers displayed lower scores in QoL scores than controls, with years of education being a significant predictor. Our results reflect a first attempt to investigate the relationships among QoL, GM volumes, and other important factors in an HD and HD caregiver sample.

**Supplementary Information:**

The online version contains supplementary material available at 10.1007/s11136-022-03220-0.

## Introduction

Huntington’s disease (HD) is an autosomal-dominant, progressive, degenerative disease characterized by multiple cognitive, motor, and behavioral difficulties [1], associated with multiple brain structural alterations. In particular, HD patients have shown early and selective atrophy in the caudate nucleus and putamen [2]. As the disease progresses, some studies have shown overall cortical thinning with larger changes in the frontal lobe, caudate, putamen and thalamus [1]. The neurocognitive and behavioral changes impact the quality of life (QoL) of patients and caregivers [3]. The QoL is considered by the World Health Organization (WHO) as “an individual’s perception of their position in life in the context of the culture and value systems in which they live and in relation to their goals, expectations, standards and concerns”. The QoL assessment usually includes four distinct domains: physical health, psychological wellbeing, social relationships, and relationship with the environment [14].

Various studies have reported QoL impairments in HD patients. For instance, Carlozzi and Tulsky [4] studied different issues that impact the QoL of HD individuals at different stages of the disease [4]. The authors found that emotional health (i.e., feelings of emotional distress and positive emotional experience) seems to be the most critical factor affecting the QoL of prodromal HD patients and caregivers. However, they also found that social health has shown to be the most relevant factor for symptomatic HD patients [4]. Other variables, such as depressive mood and impaired functional ability [3, 5], as well as executive dysfunction [3], are examples of key factors associated with decreased QoL in HD patients. Psychosocial (e.g., ability to work, symptoms of depression and anxiety), as well as cognitive (e.g., attention and problem-solving deficits), and physical aspects contributing to QoL were found to be severely impaired in symptomatic HD patients [6, 7]. Given this, although some cognitive and behavioral changes in HD patients may explain the impairments in QoL, these impairments could also be associated to structural brain changes observed in HD. However, no study has yet investigated to what extent these changes may be associated with QoL.

The QoL has been less frequently studied in caregivers than in HD patients. Even so, it is well established that caregivers’ QoL is also negatively affected by the burden of looking after a loved one with the disease [8, 9]. Particular features of HD, such as its genetic component, the extreme isolation of caregiving, and the long-term caregiving roles that may arise due to high genetic transmission, mood and satisfaction with social support are factors that strongly impact caregivers’ QoL [10, 11]. Patients’ cognitive and functional capacities seem to have the most negative effect on caregivers [12]. Considering these antecedents and the fact that previous research [10] have called for the importance of continuing assessing HD caregivers’ QoL, in this study we included a sample of caregivers of patients with HD.

The role of emotional, cognitive, and behavioral variables on HD patients’ QoL has been previously documented. The relationship between these variables and GM volume, however, has not yet been studied. Given the above antecedents, this study aimed to investigate, in HD patients, the relationship between GM volumes and QoL scores in four different domains (physical health, psychological wellbeing, social relationships, and relationship with the environment). Also, we explored which of the typical abnormalities in HD (motor dysfunction, cognitive impairment, loss of GM volume) contribute most to the QoL decrease. In addition, we assessed the QoL in a group of caregivers of HD patients and investigated whether QoL scores were associated with relevant demographic variables (sex, age, and education).

## Methods

### Study design

This study followed a case–control design aimed to study QoL in HD patients, caregivers of HD patients and healthy controls. Specifically, in HD patients, we explored the relationship between QoL scores in four different domains (physical health, psychological wellbeing, social relationships, and relationship with the environment), and (a) GM volumes, as well as (b) typical abnormalities observed in HD (motor dysfunction, cognitive impairment, loss of GM volumes). In addition, we investigated whether QoL scores of HD patients and caregivers were associated with relevant demographic variables (sex, age, and education).

### Setting

We carried out a convenience non-probabilistic sampling. Participants were contacted through a non-profit organization “Fundación Comunidades Vulnerables de Colombia” (*FUNCOVULC*). This organization aims for the integral wellbeing of people diagnosed with HD and their families. The control sample was part of the same geographic zone. All participants were selected via open invitations to participate in the study.

### Participants

We recruited seventy-five participants; 25 patients genetically and clinically diagnosed with HD, 25 caregivers of HD patients, and 25 healthy controls (Table 1). Patients were genetically and clinically diagnosed with HD, with no history of other major neurological illnesses, psychiatric disorders, or alcohol/drug abuse.

**Table 1:** Demographic, clinical, and executive function assessments

			HD (*n* = 25) Mean (SD)	CTR (*n* = 25) Mean (SD)	CRGV (*n* = 25) Mean (SD)	HD versus CRGV	HD versus CTR	CRGV versus CTR
Demographics	Age (years)		46.0 (1.84)	46.96 (12.25)	31.96 (12.14)	< 0.001	NS	< 0.001
	Sex (M:F)		9:16	15:10	6:19	NS	NS	0.009
	Education (years)		8.20 (0.57)	7.08 (4.43)	9.32 (4.161)	NS	NS	NS
QoL and clinical assessment	PDRS		74.4 (2.95)	–	98.80 (3.317)			
	WHOQOL-BREF	DOM 1	48.6 (16.55)	62.16 (8.49)	52.48 (14.65)	0.577	0.002	0.038
		DOM 2	52.08 (13.54)	66.72 (8.21)	59.36 (11.41)	0.065	< 0.001	0.061
		DOM 3	40.76 (24.67)	68.28 (17.09)	59.48 (21.11)	0.007	< 0.001	0.312
		DOM 4	41.72 (19.36)	63.64 (10.29)	48.56 (17.68)	0.303	< 0.001	0.005
		UHDRS	23.89 (9.04)	–	–			
Cognitive assessment	IFS total score		13.96 (1.12)	24.18 (1.66)	21.28 (2.88)	< 0.001	< 0.001	NS
	MOCA total score		16.20 (1.17)	27.91 (2.51)	25.68 (2.479)	< 0.001	< 0.001	NS

*HD* Huntington’s Disease Patients, *CTR* Controls, *CRGV* Caregivers, *PDRS* Physical Disability Rating Scale, *WHOQOL-BREF* World Health Organization Quality of Life scale, *UHDRS* Unified Huntington’s Disease Rating Scale, *IFS* INECO Frontal Screening, *MoCA* Montreal Cognitive Assessment

All caregivers were family members of HD patients: 40% (*n* = 10) were first-degree relatives, 48% (*n* = 12) were second-degree relatives and 12% (*n* = 3) were third-degree relatives. Only three of the caregivers did not live with his/her HD relative. Healthy controls were matched to HD patients in terms of age, sex, and years of education. Caregivers and healthy controls did not have a history of alcohol/drug abuse, HD, or other neurologic or psychiatric disorders.

All participants provided written informed consent in agreement with the Helsinki declaration. The Ethics Committee of the *Universidad Autónoma del Caribe* approved the study (*Resolution 549-A July 15, 2017*)*.*

### Variables

Our first outcome was the QoL scores in four different domains: physical health, psychological wellbeing, social relationships, and relationship with the environment. The secondary outcome variables were: GM volumes, physical disability (measured with the PDRS), and cognitive impairment (averaged scores in the MOCA test and the INECO frontal screening test). We also explored the association between QoL scores of HD patients and caregivers with relevant demographic variables (i.e., sex, age, and years of formal education).

### Data sources/measurement

The QoL was measured using the WHOQOL-BREF [14]. This scale provides a QoL profile and consists of 26 items (items 1–2 are examined separately and are not considered for the subscale scoring) assessing four different domains: physical health, psychological wellbeing, social relationships, and relationship with the environment (e.g., financial resources, safety, health and social services, opportunities to acquire new skills and knowledge, recreation, and transportation) (Supplementary file, S1). The WHOQOL-BREF does not provide an overall score, only an individual score for each domain. In each domain, scores range from 0 to 100 and are scaled in a positive direction (higher scores indicate a better QoL). We chose this instrument based on several reasons. First, this is a tool designed cross culturally by the World Health Organization (WHO), assessing multiple key generic QoL domains (physical health, psychological wellbeing, social relationships, and relationship with the environment) which are relevant for multiple clinical and non-clinical populations, including HD patients. Second, the WHOQOL-BREF has good to excellent psychometric properties of reliability and performs well in tests of validity [15]. Third, this questionnaire is suitable for QoL assessment not only in HD patients, but also in caregivers and healthy participants. It is worth noting that, although the WHOQOL-BREF was not specifically designed to assess QoL in HD, it has been used previous studies in neurodegenerative diseases [16, 17], including HD patients [18, 19]. This instrument has also been employed to assess QoL in caregivers of HD patients [11] and healthy individuals [20, 21].

The participants’ general cognitive state and executive functioning were assessed with the Montreal Cognitive Assessment (MOCA) (see Supplementary file, S2) and the INECO Frontal Screening (IFS) (see Supplementary file, S3), respectively. Both instruments have been widely used and have proven to be suitable tools in the cognitive assessment of neurodegenerative diseases [22–24]. We assessed cognitive functioning due to the prevalence of cognitive impairment in HD population, as well as its significant impact on quality of life, beyond motor and psychiatric symptoms [25].

Patients with HD underwent a neurological examination and were assessed using the Unified Huntington’s Disease Rating Scale (UHDRS) and the Physical Disability Rating Scale (PDRS). (Table 1). PDRS scores have been used in other studies interested in HD [26]. Also, the UHDRS is the predominant instrument in assessing comprehensively HD’s impact on functionality [27]. The PDRS measures functionality on a 10–100 scale. Measures increase by ten, being 10 the lower score and meaning total dependence on external aids. A score of 100 indicates normal functionality, no apparent illness.

### Bias

Significant differences in age and sex were found between caregivers and controls as well as between patients and caregivers. Considering these differences, age was included as a covariate for all behavioral comparisons between among groups. Both, age and sex, were included as predictors in multiple regression analyses for caregivers.

### Statistical methods

#### Behavioral results

The QoL scores were compared using one-way ANOVAs and Tukey’s HSD post-hoc tests, when necessary. To control for the influence of age on the QoL ratings, ANCOVA tests were applied to compare (a) patients vs. caregivers and (b) caregivers vs. controls. Effect sizes were calculated through partial eta squared (*η*^2^). Following Cohen’s classification of effect sizes [28], we considered effects to be statistically relevant at *η*^2^ ≥ 0.01 (i.e., small or higher effect sizes).

A post-hoc sensitivity analysis was performed using G*power [29]. Results of this analysis showed that, assuming a power of 0.80 and *α* level of 0.05, our sample size was sufficient to detect a medium/large effect size (*f* = 0.36, critical *F* = 3.12).

#### VBM (Voxel-based morphometry analyses)

Images were preprocessed using the DARTEL Toolbox, in accordance with previously described procedures [30] (Supplementary file, S6). Using SPM-12, we compared the GM volumes of (a) patients vs. the control group, (b) patients vs. caregivers and (c) caregivers vs. controls. For these analyses, total intracranial volume (TIV) was included as a covariate of no interest (*p* < 0.001 uncorrected, extent threshold > 30 voxels).

#### Relationship between GM volumes and HD patients’ QoL

Multiple regression analyses were performed to explore the association between GM volumes of HD patients and scores in each one of the QoL domains. The TIV was included as a covariate of no interest in all analyses (*p* < 0.001 uncorrected, extent threshold > 30 voxels).

#### Other relevant factors associated with QoL

For the group of HD patients, we used multiple regression analyses to explore the association between each QoL score and (a) GM volume in areas related to scores in each domain, (b) physical disability, or (c) general cognitive state and executive function. Four different models were estimated. We included cognitive functioning in the regression models due that impairments in general cognitive [31] and executive functions [32] are frequent in HD patients, as well as their significant impact on QoL beyond motor and psychiatric symptoms [12, 25]. Also, physical disability scores were included as predictor in the models given that it has been suggested that motor symptoms correlated positively with functional ability and in turn, physical disability correlates significantly with QoL decline in HD patients [33]. However, the specific contribution of physical disability to QoL impairment has not been further explored in HD patients. Scores on each QoL domain were independently considered as dependent variables for these models. For all models, the following predictors were considered: The GM volume values from the clusters significantly associated with each domain, the PDRS ratings, and an overall cognitive functioning index (average of an individual patient’s MOCA and IFS scores). The MOCA and IFS ratings were averaged to diminish the number of predictors contained in each model.

We conducted a second set of multiple regressions for caregivers and controls to explore whether their QoL scores were associated with (a) the group (being caregivers or controls), or (b) relevant demographic variables. Scores on each QoL domain were independently considered as dependent variables for these models, and group, sex, years of formal education, and age were considered as predictors. The statistical significance level was set at *p* < 0.05.

## Results

### Behavioral results

**Fig. 1:**
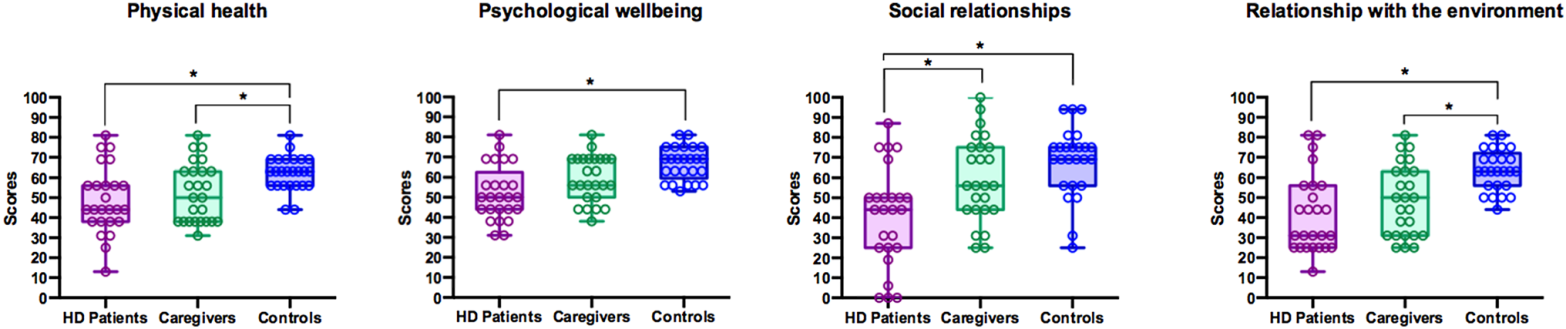
Group comparisons of QoL scores. Asterisks indicate significant differences among groups

Figure 1 shows group comparisons on QoL scores. Significant differences were observed among groups in the physical health domain (*F*(2,72) = 6.526; *p* = 0.002; *η*^2^ = 0.153). Post-hoc analyses (Tukey’s HSD, MS = 1219.3; *df* = 72) showed that patients (*p* = 0.002) and caregivers (*p* = 0.038) scored significantly lower than controls. No significant differences were found between patients and caregivers (*p* = 0.577). The analysis of covariance showed that differences between caregivers and controls (*p* = 0.003) remained significant after controlling for age (*p* = 0.704).

Analysis of the psychological domain also showed significant differences among groups (*F*(1,72) = 10.55; *p* < 0.001; *η*^2^ = 0.227). Post-hoc analyses (Tukey’s HSD, MS = 1339.6; *df* = 72) showed that patients’ scores were significantly lower than those of controls (*p* < 0.001), but not of caregivers (*p *= 0.064). No significant differences were found between caregivers and controls (*p* = 0.061). A covariance analysis showed that differences between patients and caregivers (*p* < 0.001) could not be explained by age (*p* = 0.247).

Results in the social relationships domain showed significant differences among groups (*F*(1,72)  = 11.00; *p* < 0.001; *η*^2^ = 0.234). Post-hoc analyses (Tukey’s HSD, MS = 4938.5; *df* = 72) revealed that patients’ ratings were significantly lower than controls (*p* < 0.001) and caregivers (*p* = 0.007). No significant differences were found between caregivers and controls (*p* = 0.312). The analysis of covariance showed that these differences between patients and caregivers (*p* < 0.001) prevailed after controlling for age (*p* = 0.247).

Finally, scores in the environment domain showed significant differences among groups (*F*(2,72)  = 11.89; *p* < 0.001; *η*^2^ = 0.248). Post-hoc analyses (Tukey’s HSD, MS = 3144.5; *df* = 72) showed that patients scored significantly lower than controls (*p* < 0.001) but caregivers did not (*p* = 0.303). In addition, significant differences were found between caregivers and controls (*p* = 0.005). The analysis of covariance showed that these differences between caregivers and controls (*p* < 0.001) prevailed after controlling for age (*p* = 0.270).

In sum, patients’ ratings in all four domains were significantly lower than those of controls. Caregivers scored significantly lower than controls in physical health and the environment domains. These differences remained significant after controlling for age.

### VBM results

#### HD brain atrophy

**Fig. 2:**
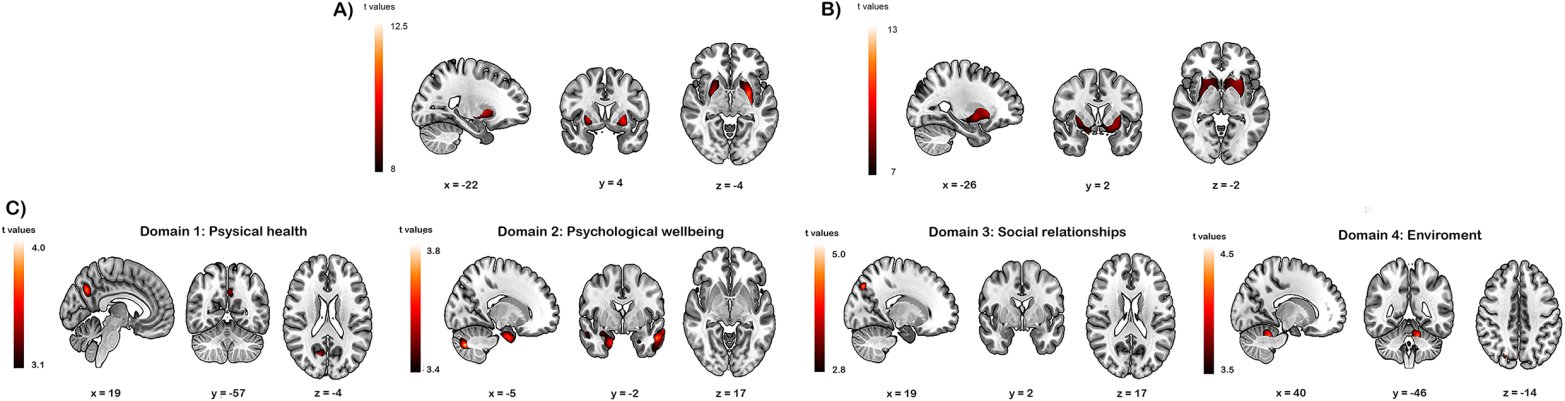
**A** Regions of significant gray matter volume loss in the HD group compared with the control group (*p* < 0.001, uncorrected). **B** Regions of significant gray matter volume loss in the HD group compared with the caregiver group (*p* < 0.001, uncorrected).** C** Significant associations between GM volumes and QoL scores in patients with HD (*p* < 0.001, uncorrected)

Compared to controls, HD patients showed reduced GM volumes in the right and left putamen, the left cerebellum, and the medial frontal gyrus (Fig. 2A). When comparing patients and caregivers, HD patients showed reduced GM volumes in the right supramarginal gyrus, the right and left putamen, and the left cerebellum (Fig. 2B). As expected, comparisons between the control group and caregivers showed no suprathreshold clusters.

#### Brain regions associated with QoL in HD patients (see Table 2)

**Table 2:** Association between gray matter volumes and subscores for each dimension of QoL

QoL Domain	Brain regions	Peak T	*Z* value	Cluster size	Coordinates *x*, *y*, *z*		
DOM 1	Left precuneus	3.66	3.22	64	− 5	− 63	35
DOM 2	Middle temporal gyrus	5.29	4.24	3015	− 52.5	1.5	− 25.5
	Right cerebellum	5.29	4.24	3795	18	− 70.5	− 39
	Left cerebellum	4.30	3.64	841	− 37.5	− 64.5	− 28.5
	Inferior occipital gyrus	4.11	3.52	58	− 30	− 90	− 22.5
	Precuneus	3.87	3.36	123	18	− 69	45
	supramarginal gyrus	3.87	3.36	82	54	− 43.5	33
DOM 3	Precuneus	5.01	4.07	534	22.5	− 72	37.5
	Inferior occipital gyrus	4.15	3.55	294	31.5	− 85.5	− 21
	Precuneus	4.01	3.46	124	21	− 69	43.5
	Right cerebellum	3.85	3.35	562	13,5	− 54	− 21

Figure 2C shows the significant associations between GM volumes and QoL scores in patients with HD.

*Physical health* Patients showing lower GM volumes in the left precuneus recorded lower scores on this domain.

*Psychological wellbeing* Reduced GM volumes in the middle temporal gyrus, the right supramarginal gyrus, the inferior occipital gyrus, the right precuneus and the right and left cerebellum were associated with lower scores in this domain.

*Social relationships* Patients showing reduced GM volumes in the precuneus recorded lower scores in the social relationships domain.

*Relationship with the environment* Reduced GM volumes in the right cerebellum, the inferior occipital gyrus and the precuneus were associated with lower scores in this domain.

### Relationships among QoL, GM volumes, physical disability, and overall cognitive function

#### Patients

For the first three domains as dependent variables (physical health, psychological wellbeing, and social relationships), it was found that all variables (i.e., GM clusters associated with each domain, overall cognitive function, and PDRS scores) were significant predictors (Table 2). The fourth model, which included relationship with the environment scores as a dependent variable [*F*(3,14) = 6.439, *p* = 0.009, *R*^2^ = 0.637], showed that only the GM volumes associated

with this domain (*p* = 0.010; *β* = 0.654) and the PDRS scores (*p* = 0.019; *β* = −0.559) were significant predictors (Table 3).

**Table 3:** Coefficients of the multiple regression models of the relationship between GM volumes, cognition, disability and QoL (patients)

Variables	Model I: Physical health				Model II: Psychological				Model III: Social relationships				Model IV: Environment			
	*β*	*p*	95% CI		*β*	*p*	95% CI		*β*	*p*	95% CI		*β*	*p*	95% CI	
			Lower	Upper			Lower	Upper			Lower	Upper			Lower	Upper
Atrophy Index	0.462	0.038	9.629	280.105	0.718	0.037	19.264	503.618	0.679	0.012	0.475	5.951	0.654	0.010	− 0.117	3.635
Cognitive Performance Index	0.525	0.035	0.205	4.622	0.922	0.019	0.472	4.275	0.643	0.025	81.926	518.557	0.463	0.063	109.520	641.351
PDRS	− 0.648	0.013	− 1.855	− 0.276	− 0.745	0.021	− 1.247	− 0.124	− 0.546	0.034	− 1.865	− 0.088	− 0.559	0.019	− 1.368	−0.151

*PDRS* Physical Disability Rating Scale

### Relationships among QoL, gender, age, years of education and group

#### Caregivers and controls

Years of formal education were a significant predictor for all four domains (Table 4). Group was found to be a significant predictor for three domains: psychological wellbeing, social relationships and relationship with the environment (Table 4). Neither sex nor age were found to be significant predictors of QoL.

**Table 4:** Coefficients of the multiple regression models of the relationship between demographical variables and QoL (caregivers and controls)

Variables	Model I: Physical health				Model II: Psychological				Model III: Social relationships				Model IV: Environment			
	*β*	*p*	95% CI		*β*	*p*	95% CI		*β*	*p*	95% CI		*β*	*p*	95% CI	
			Lower	Upper			Lower	Upper			Lower	Upper			Lower	Upper
Gender	0.128	0.389	− 4.327	10.893	− 0.012	0.922	− 5.656	5.129	0.109	0.428	− 6.490	15.038	− 0.128	0.348	− 13.004	4.680
Group	− 0.336	0.055	− 17.264	0.186	− 0.430	0.005	− 15.145	− 2.779	− 0.331	0.042	− 25.145	− 0.462	− 0.542	0.001	− 27.542	− 7.266
Age	0.050	0.796	− 0.301	0.390	0.146	0.381	− 0.137	0.353	− 0.053	0.766	− 0.562	0.416	0.106	0.550	− 0.282	0.522
Level of education	0.109	0.520	− 0.669	1.304	0.583	< 0.001	0.695	2.093	0.448	0.006	0.593	3.383	0.318	0.045	0.027	2.319

## Discussion

The primary objective of this study was to explore, in HD patients, the relationship between GM volumes and QoL scores in four different domains (physical health, psychological wellbeing, social relationships, and relationship with the environment). As secondary objective, we aimed to assess which of the typical abnormalities in HD (motor dysfunction, cognitive impairment, loss of GM volume) contribute most to the QoL decrease. We also we assessed the QoL in a group of caregivers of HD patients and investigated whether QoL scores were associated with relevant demographic variables (sex, age, and years of education). Compared to controls, HD patients showed significantly lower scores in all the QoL domains. Compared to caregivers, patients scored significantly lower only in the social relationships’ domain. Caregivers scored significantly lower than controls in the physical health and the environment domains. Additionally, in HD patients, lower scores in QoL domains were associated with low GM volumes, mainly in the precuneus and the cerebellum. These results reflect a first attempt to investigate relationships among QoL, GM volumes, and other important factors involved in HD.

### Behavioral results

Consistent with previous reports, our results showed that HD patients scored lower than caregivers and controls in all four domains of the WHOQOL-BREF [34]. Physical health domain encompasses multiple subdomains, such as work capacity, mobility, sleep, rest, and dependence on medicinal substances and medical aids. Consistent with our results, previous studies have shown that work capacity [6] and mobility [35] are highly affected in HD-diagnosed patients, and even could be affected in the pre-diagnostic phase [36]. Psychological wellbeing examines areas such as self-esteem, positive and negative feelings, and body image and appearance. Consistent with our findings, previous studies have reported that the psychological wellbeing of HD patients is highly compromised [6], even when compared to patients with other neurodegenerative diseases (i.e., Parkinson’s disease and multiple sclerosis) [17].

Also, our results showed that HD has a negative impact on social relationships (affecting satisfaction levels with sex life), perceived support from external social networks, and satisfaction with personal relationships. In line with this result, it has been shown [37] that individuals with manifest HD show low levels of satisfaction and perceived ability to participate in social roles and activities. Previous research has also shown that sexuality is highly altered in HD, as hypoactive sexual behavior is the most common sexual disorder [38].

The relationship with the environment describes an individual’s perception of opportunities to participate in different settings, receive professional assistance, and enjoy physical environments, among others. Limited financial aid/specialized services account for most of the difficulties caregivers and HD patients face with the environmental factor. It has already been established that, due to the particularities of the disease, caregivers and patients seldom gain access to specific services and trained professionals able to deal with such problems [13]. These factors account for the significantly lower scores patients and caregivers have compared to controls in the relationships with the environment domain. Furthermore, patients scored significantly lower than caregivers. It is possible that common behavioral symptoms (disinhibition, depressed mood, euphoria) impede to an even greater degree the capacity of HD patients to be an active part of their surroundings.

Regarding caregivers, Pino et al. [34] also found significant differences between caregiver and control scores in the physical health domain, suggesting that taking care of a relative with the disease has implications in areas such as perceived energy and fatigue, amount of sleep, and rest/work capacity. This is in line with findings of Aubeeluck et al. [10] that showed caregivers often feel tired, exhausted, and overwhelmed while caring for an HD relative at the same time as their children and themselves.

### VBM results

Our results showed that compared to controls, HD patients exhibited reduced GM volumes in the right and left putamen, the left cerebellum, and the medial frontal gyrus. These results are consistent with the atrophy pattern previously reported in HD [36, 39, 40].

For the relationship between GM volume and QoL scores in HD patients, we found that low GM volumes in the precuneus were associated with low scores in the four assessed domains. The precuneus is known to be involved in multiple functions such as episodic and autobiographical memory, self-awareness, self-reflection, introspection, and social cognition [3, 41, 42]. Also, as HD progresses, the precuneus is one of the most affected regions [43]. A previous study [44] of the general population reported an association between GM volume in the precuneus and physical/general health. Consistent with our results, previous HD studies have shown associations between social cognition abnormalities (i.e., reduced empathy and social emotions) and the precuneal GM volume [26] or activity [45]. Considering precuneus general involvement in multiple cognitive processes and its progressive neurodegeneration in HD, it is consistent to find that lower GM volumes in this area correlate with lower QoL in HD patients.

We also found that lower GM volumes in the cerebellum were associated with lower scores in the psychological wellbeing and the relationship with the environment domain in HD patients. Atrophy of the cerebellum starts in early stages of HD and involves multiple neurodegenerative features [42, 46]. Given that cerebellar atrophy is associated with classical HD symptoms (i.e., ataxia, delay in the initiation and termination of movements, hypotonia, dysarthria, and impaired fine movements) [46], it is to be expected that awareness of these difficulties could lead to negative self-feelings and lower self-esteem, affecting the QoL in the psychological wellbeing domain. Furthermore, because of the progression of motor symptoms, available external resources become scarce, and home environments may have to be greatly accommodated, also negatively affecting the evaluation of the environment.

Moreover, lower GM volumes in the supramarginal, the middle temporal, and the inferior occipital gyri were associated with lower scores in the psychological wellbeing domain. Consistent with these results, a recent study [47] showed that, compared to healthy controls and HD patients without dementia, HD patients with dementia exhibited a prominent pattern of GM reduction restricted to regions of the parietal, temporal, and occipital cortices, including the supramarginal, the middle temporal gyrus and the inferior occipital gyri. Consistent with our results, these brain alterations were associated with poorer cognitive performance in HD [47–49]. It is expected, then, that lower GM volumes in these brain regions correlate with lower scores in psychological wellbeing, given that memory, learning, and thinking are some of the capacities in this domain. Impairments in these cognitive domains likely affect the psychological wellbeing of HD patients.

### Relationship between QoL, GM volumes, physical disability, and overall cognitive function

For HD patients, we found that PDRS scores and GM volumes in the regions associated with QoL predicted scores in all domains. This suggests that atrophy in regions associated with cognitive, social, and motor functions, as well as the severity of physical disability, are key determinants of QoL perception in our sample. Consistent with these results, Helder et al. [6] found that physical symptomatology affected QoL in psychological and physical dimensions, although explaining a larger amount of variance for the latter. Moreover, functionality has been previously proposed [50] as an important predictor of QoL for HD patients.

General cognitive function was significantly associated with three of the four domains: physical health, psychological wellbeing, and social relationships. This may be explained by the patient’s awareness of impairments in these domains, as well as by the direct consequences of cognitive impairment. As mentioned, functional and cognitive incapacities have the most detrimental effects on the QoL of HD patients [12]. This highlights the importance of disease-specific interventions that target cognitive impairment and functionality to improve QoL in this population. It is worth noting that cognitive function did not predict the relationships with the environmental domain. This result could be explained because the items measured entail primarily factors that surpass the patient’s control. The variables measured here correspond to external factors, for example, financial aid. Having an altered cognitive functionality has little influence on receiving economic aid or having streets in good condition, for example.

Results for caregivers showed that years of formal education predicted for all domains. This could be explained by the fact that higher education enables the individual to have a better job and so fulfill more easily and consistently all his/her individual and family needs. Moreover, we found that being a caregiver, in comparison to being a control, is also associated with QoL scores in our sample. It has been established [13] that caregivers of HD deal with moods and behavioral changes that significantly alter family dynamics. Aubeeluck et al. [10], suggest that there is a need to consider HD caregiving independently due to some unique factors of the disease: caring for dementia compounded by extreme isolation, and the possibility of being the caregiver for multiple generations due to the genetic characteristics of the disease, resulting in long-term caregiving roles. Our results showed that neither sex nor age were significant predictors of QoL in caregivers. This potential association had not been previously assessed. However, studies on QoL in caregivers of patients with other neurodegenerative diseases have shown that being a female caregiver correlates with greater caregiving burden [51]. Also, studies interested in the association between QoL and demographic variables have highlighted the importance of age and sex in understating QoL of different populations [52–54]. Thus, future research should further investigate the association between demographical variables and QoL in caregiver of HD patients.

### Limitations

Several limitations must be recognized. First, Although our sample size was relatively small, it is similar to that of previous studies assessing GM atrophy [1, 55, 56], or QoL [3, 12] in HD patients or QoL in caregivers of HD patients [9, 57]. Second, even though we assessed several key variables previously associated to QoL in HD patients (demographic variables, cognitive measurements, and physical disability outcomes) [27, 32, 33], as well as GM volumes, other relevant variables such as behavioral and psychiatric symptoms such as depression, suicidal ideation, anxiety, irritability, anger/aggression, apathy, perseveration and others [58] were not considered in the current study. Third, although we measured QoL with a standard and validated measure [59], there have been developed specific QoL questionnaires for HD population and their caregivers (e.g., Mestre et al. [60]). Fourth, there were significant differences in important demographic variables (sex and age) between our study and control groups. Finally, our sample was chosen by convenience, there was non-random sampling for this investigation.

### Conclusion

In conclusion, our results suggest GM volume and physical disability are the most important predictors of QoL decline in HD patients. In particular, brain regions involved in diverse cognitive, social, and motor functions (precuneus and cerebellum) displayed the greatest relationships with QoL measures. Additionally, we showed that HD caregivers display lower levels of QoL than controls, and that years of education was the most important predictor. Overall, this study is the first to correlate QoL measures and neural substrates in HD. This work will enable further investigation to better comprehend the impact of this illness in multiple adjustment areas, encouraging more disease-specific prevention strategies, treatments, and aids for patients and their caregivers.

## Supplementary Information

Below is the link to the electronic supplementary material.

Supplementary file1 (DOCX 30 KB)
