# Supplementary material for: Brain, cognitive, and physical disability correlates of decreased quality of life in patients with Huntington’s disease
Source: Qual Life Res. 2022 Aug 17;32(1):171–82. doi: 10.1007/s11136-022-03220-0 (PMC9829572; doi:10.1007/s11136-022-03220-0)
Supplement: Supplementary file 1 — Supplementary file1 (DOCX 30 KB) [file 11136_2022_3220_MOESM1_ESM.docx]

**Supplementary File**

**S1. Quality of life measurement.** The WHOQOL-BREF is a shorten version of the WHOQOL-100. This brief version assesses quality of life in four principal domains: physical health, psychological, social relationships and environment. The questionnaire is made up of 26 questions; the first two questions ask for the overall quality of life and general health; the other 24 questions make up the domains. Domain scores are scaled in a positive direction. The WHOQOL-BREF domain’s scores can be interpreted in two manners: converting them to a 4-20 scale or to a 0-100. For this study, scores were converted to the second scale. The following are the evaluated aspects in each domain and can be found in:

| **Domain** | **Facets incorporated within domains** |
| --- | --- |
| 1. Physical health | Activities of daily living  Dependence on medicinal substances and medical aids  Energy and fatigue  Mobility  Pain and discomfort  Sleep and rest  Work capacity |
| 1. Psychological | Bodily image and appearance  Negative feelings  Positive feelings  Self-esteem  Spirituality/Religion/Personal beliefs  Thinking, learning, memory and concentration |
| 1. Social relationships | Personal relationships  Social support  Sexual activity |
| 1. Environment | Financial resources  Freedom, physical safety and security  Health and social care: accessibility and quality  Home environment  Opportunities for acquiring new information and skills  Physical environment (pollution / noise / traffic / climate)  Transport |

**S2.** The Montreal Cognitive Assessment is a screening device used to assess several cognitive domains. It is made up of eight subsections. The first measures visuospatial abilities and cognitive functioning. The second evaluates short-term memory with a list of five words, which will be asked at the seventh subsection. The third subtest evaluates attention and working memory. The fourth, evaluates language; the fifth tests for abstraction capacities and the eight consists of five questions to evaluate orientation. The maximum is 30. For patients with less that 12 years of education, an additional point is given to the final score. Scores of 25 or less indicate cognitive dysfunction.

**S3.** The INECO Frontal Screening (IFS) consists of eight subtests: (1) motor programming (Luria series, “fist, edge, palm”); (2) conflicting instructions (hitting the table once when the administrator hits it twice, or hitting it twice when the administrator hits it only once); (3) motor inhibitory control; (4) numerical working memory (backward digit span); (5) verbal working memory (months backwards); (6) spatial working memory (modified Corsi tapping test); (7) abstraction capacity (inferring the meaning of proverbs), and (8) verbal inhibitory control (modified Hayling test). The maximum possible score on the IFS is 30 points. This test has been shown to successfully detect executive dysfunction in patients with neurological and psychiatric diseases.

**S4.** **MRI scanning**. A T1-weighted spin echo sequence was used to generate contiguous axial slices covering the whole brain (TR=7489 ms; TE=3420 ms; flip angle=8°; FOV= 256x256 mm; matrix size=256x240; 1mm isotropic).

**S5. VBM analysis.** Images were preprocessed using the DARTEL Toolbox, in accordance with previously described procedures. Then, modulated 12-mm full-width half-maximum kernel-smoothed images were normalized to the MNI space and analyzed through general linear models for 2nd level analyses on SPM-12 software. To explore regional GM reduction in the HD group and caregivers relative to controls, a two-sample comparison, including total intracranial volume (TIV) as a confounding covariate (*p* < 0.01, not FWE corrected, extent threshold=30 voxels), was performed.
